# Optimization of Fluorometric Assay for Ozone in Solution

**DOI:** 10.1155/2014/190937

**Published:** 2014-03-24

**Authors:** Hiromi Nagano, Gotaro Shiota, Hidetoshi Arakawa

**Affiliations:** Department of Analytical Biochemistry, School of Pharmacy, Showa University, 1-5-8 Hatanodai, Shinagawa-ku, Tokyo 142-8555, Japan

## Abstract

Ozone is widely used for sterilization, deodorization, and cleaning. However, the effects of ozone on human are not well understood, because there is currently no reliable assay method for ozone. Therefore the accurate measurement of ozone is important for its safe use. Here, we report optimized conditions for use with fluorescein derivative **1** (probe **1**). Under optimum conditions, probe **1** reacted with ozone quantitatively and specifically to generate a fluorescent substance with an excitation maximum at 493 nm and an emission maximum at 523 nm. We conclude that this assay will be useful for quantifying ozone in solution.

## 1. Introduction

Ozone is used for sterilizing fingers, foods, and tap water for preventing nosocomial infection and deodorization. Recently, ozone has been used in cosmetics, for sterilization after dental treatment and for washing the skin around burns.

However, the effects of ozone on human are unknown, and there is currently no reliable method to evaluate its effects. Thus, prior to the application of ozone in medical care, a means for accurately measuring ozone is required.

Ozone is currently measured by the ultraviolet absorption [1], the iodine titration [2], the indigo dye [3, 4], or the galvanic method [5]. However, these methods are not sensitive and specific for ozone.

**Figure 1:**
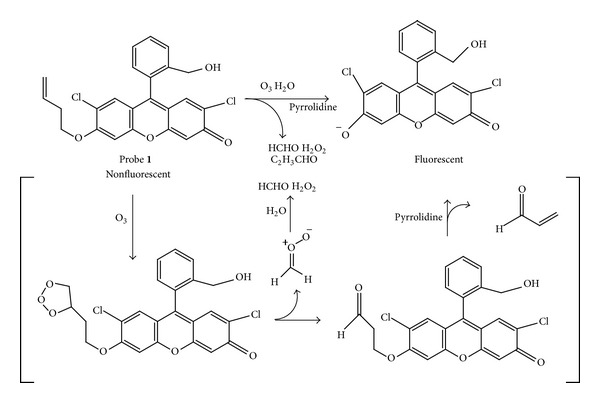
Reaction of probe** 1** with ozone.

The Koide group previously reported probe** 1** and demonstrated proofs of concepts (Figure 1) [6]. However, the use of probe** 1** was not specifically optimized for quantifying ozone in solution. Given the paucity of user-friendly, selective, and robust method for measuring ozone in solution, we set out to validate and optimize the fluorometric method with probe** 1**. We report herein the optimum conditions for measuring ozone using probe** 1**.

## 2. Materials and Methods

### 2.1. Reagents

Reagents were obtained from the following suppliers: hydrogen peroxide 30%, iron (II) sulfate heptahydrate, NaClO, and hypoxanthine that were from Wako Pure Chemical Industries Ltd. (Osaka Japan). Xanthine oxidase was obtained from bovine milk and pyrrolidine from SIGMA-ALDRICH, Co (USA). Diethylenetriaminepentaacetic acid (DETAPAC) and NOC-7 were obtained from DOJIN MOLECULAR TECHNOLOGIES, INC. All other chemicals were obtained as reagent-grade.

Probe 1 was donated kindly from Professor K. Koide of University of Pittsburgh [6].

Ozone-containing water was prepared by the electrolysis of water (ozone generator: VMC JAPAN). The concentration of standard ozone-containing water was calculated from its absorbance at a wavelength of 254 nm using a molar absorptivity coefficient, *ε*, of 2950 mol^−1^Lcm^−1^ [7].

Preparation of probe 1 solution for assay: a solution of probe** 1 **(12.5 *μ*mol/L) in a mixture of 5% v/v methanol in a 0.5 mmol/L phosphate buffer (pH 3.3) was prepared immediately prior to use.

### 2.2. Preparations of OH Radical, Superoxide, and Nitric Oxide

Conditions for generation of OH radical (the Fenton reaction): 0.5 mmol/L phosphate buffer (pH 7) 1600 *μ*L, 5 × 10^−4^ mol/L FeSO_4_ solution 300 *μ*L, 1 × 10^−3^ mol/L DETAPAC 300 *μ*L, H_2_O 500 *μ*L, and 400 ppm H_2_O_2_ 300 *μ*L [8].

Conditions for generation of superoxide anion radical (•O_2_^−^): 40 ppm hypoxanthine 90 *μ*L and 0.1 U/mL xanthine oxidase 10 *μ*L were mixed [9].

Condition for generation of nitric oxide (NO): 1 mg/mL NOC-7 50 *μ*L was diluted with H_2_O 1200 *μ*L [10].

### 2.3. Method

Ozone was measured as follows. A sample of ozone-containing water (100 *μ*L) was added to the solution of probe** 1** (900 *μ*L) and the resulting solution was incubated at 37°C for 1 h. Then 50 mmol/L pyrrolidine solution (100 *μ*L) was added to complete the *β*-elimination in 5 minutes room temperature. Upon the completion of the elimination, the fluorescence intensity was measured (ex. 493 nm, em. 523 nm).

### 2.4. Apparatus

Spectrophotometer (JASCO V-530, JAPAN) for UV measurement of ozone concentration and spectrofluorometer (JASCO FP-6500, JAPAN) for measurement of fluorescence of probe 1 were used.

## 3. Results and Discussion

Specifically, we examined the effects of pH, reaction time, and the probe concentration, to develop a highly sensitive and accurate method for measuring ozone.

**Figure 2:**
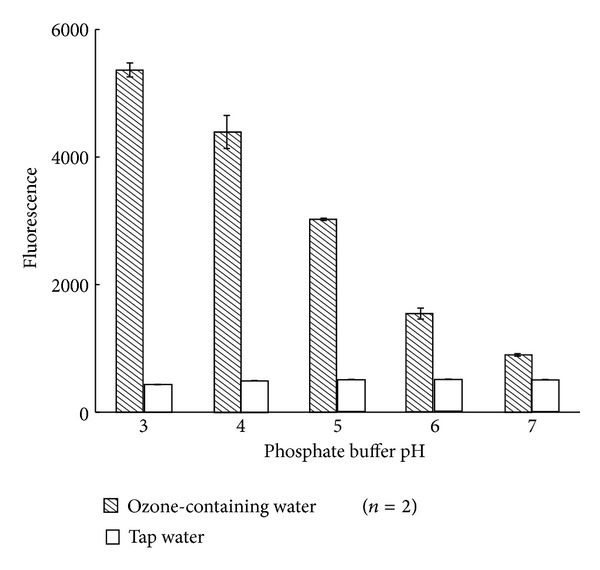
Effect of phosphate buffer pH on fluorescence intensity.

First, the effects of the pH values of 5 mmol/L phosphate buffers on fluorescence intensity were studied. As shown in Figure 2, the fluorescence intensity and signal-to-noise (S/N) ratio increased as the pH decreased. As a result, we chose pH 3 as the optimum pH. Generally the decomposition of ozone in aqueous solution is catalyzed by OH^−^ ions [11]. Therefore, the acidic condition stabilize ozone, and it promotes ozonolysis of probe** 1 **in the first step.

**Figure 3:**
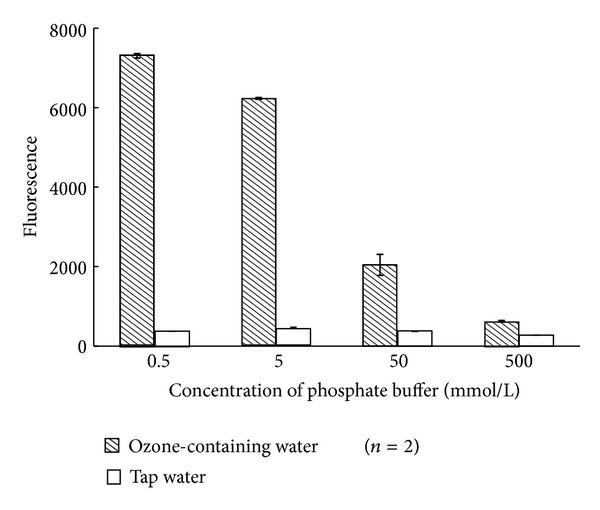
Effect of concentration of phosphate buffer on fluorescence intensity.

The effect of the salt concentration of the pH 3 phosphate buffer on fluoremetric method was investigated. As shown in Figure 3, the fluorescence intensities and S/N ratios increased as the concentrations of salt decreased. Therefore, we chose 0.5 mmol/L for the following experiments.

**Figure 4:**
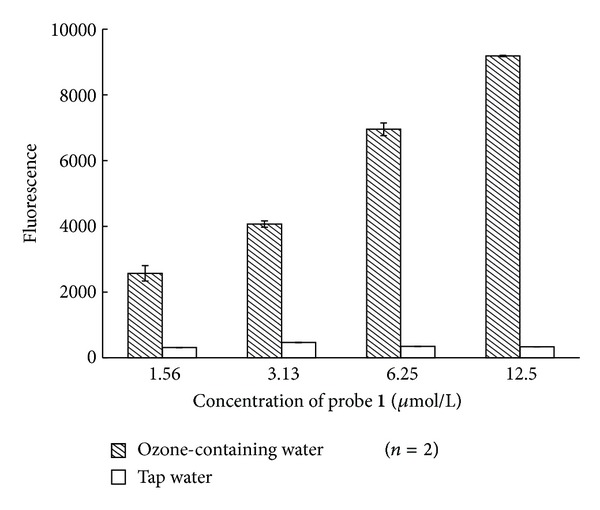
Effect of concentration of probe** 1** on fluorescence intensity.

Next, probe** 1** was examined at 1.56 *μ*mol/L, 3.13 *μ*mol/L, 6.25 *μ*mol/L, and 12.5 *μ*mol/L concentrations. The fluorescence intensity and S/N ratio were the highest at 12.5 *μ*mol/L, as shown in Figure 4. Because the kinetics of alkene ozonolysis is first-order with respect to an alkene [6], this observation is consistent with the literature.

**Figure 5:**
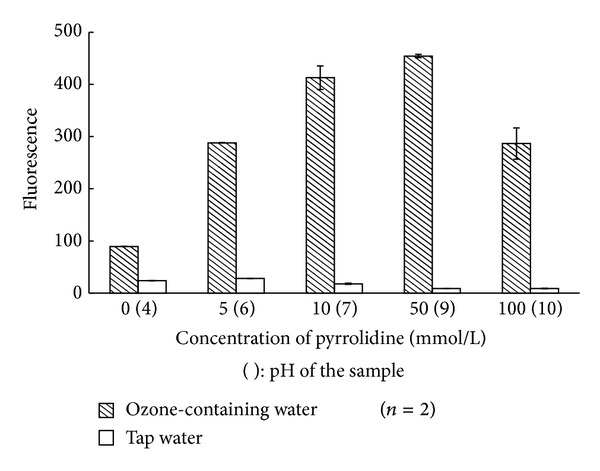
Effect of concentration of pyrrolidine on fluorescence intensity. (): pH for fluorometry.

The concentration (0–100 mmol/L) of pyrrolidine solution used for catalysis was determined. The fluorescence intensity of ozone water increased as the concentration of the pyrrolidine solution increased, although a decrease was eventually observed: a decrease in the fluorescence intensity of ozone water was observed between 50 and 100 mmol/L, and for the blank between 5 and 10 mmol/L, as shown in Figure 5. We chose 50 mmol/L as the optimum concentration of pyrrolidine because this provided the strongest fluorescence intensity and largest S/N ratio. The increase in the fluorescence intensity may arise from promotion of aldehyde *β*-elimination [12].

**Figure 6:**
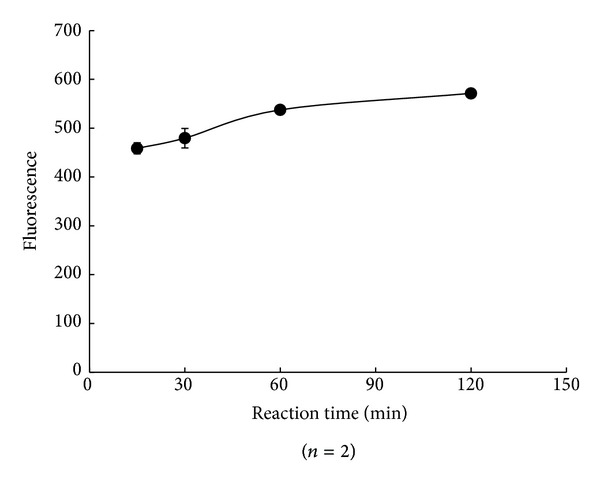
Effect of reaction time at 37°C on fluorescence intensity.

The reaction time (15, 30, 60, or 120 min at 37°C, or overnight (120 min at 37°C, then 4°C)) was determined. The fluorescence intensity and S/N ratio were strongest at 120 min, but there was little difference between 60 min and 120 min, as shown in Figure 6. And the S/N ratio decreased overnight (data not shown). Therefore, we chose 60 min as the optimum reaction time.

**Figure 7:**
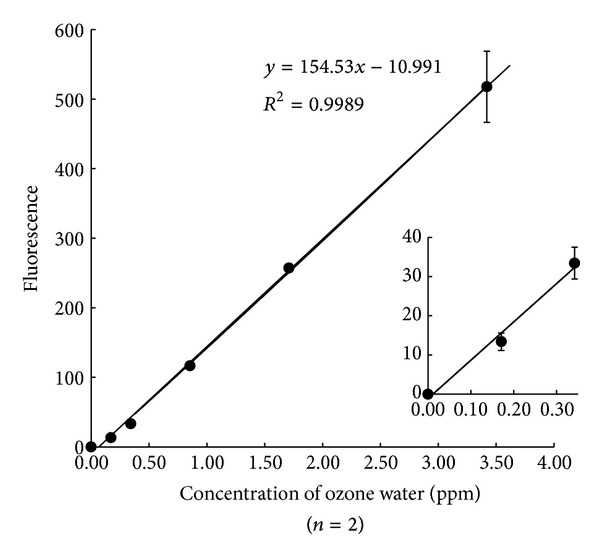
Standard curve for ozone.

A standard curve for ozone water is shown in Figure 7. The limit of detection of ozone in water was 0.49 pmol from blank + 3SD.

The reproducibility of this analysis was then investigated using tap water as the blank: CV = 0.9%, *n* = 7; Ozone-containing water 0.16 ppm: CV = 2.66%, *n* = 7; Ozone-containing water 3.25 ppm: CV = 4.82%, *n* = 7. These data show satisfactory reproducibility of this assay.

The specificity of this assay was also determined. Hydroxyl radical (OH) from the Fenton reaction was used. Hydroxyl radical, NaClO (40 ppm), O_2_^−^ radical from xanthine oxidase, NaNO_2_ (40 ppm), KNO_3_ (40 ppm), nitric oxide from NOC-7, and hypoxanthine were also assessed. The following fluorescence intensities were obtained: tap water: 0.38%, NaClO: 0.30%, OH radical: 0.29%, O_2_^−^: 0.43%, NO: 0.40%, NO_2_^−^: 0.36%, NO_3_^−^: 0.38% when the radical fluorescence intensity of ozone water (6.3 ppm) was 100% (*n* = 2). Therefore, Probe** 1** has high specificity for ozone.

**Figure 8:**
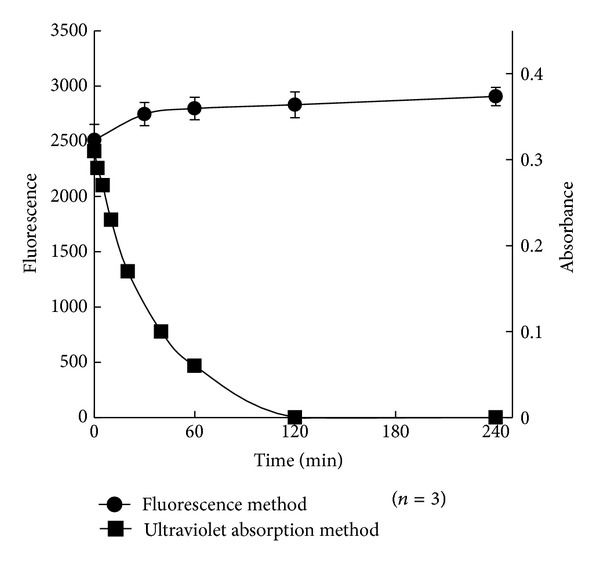
Stability of the reaction product from Probe** 1** reacted with ozone (fluorescence method) and absorbance of ozone water (ultraviolet absorption method).

Finally, the stability of the product formed by the reaction of probe** 1** with ozone was investigated by fluorescence intensity. After pyrrolidine solution was added and reacted for 5 min at room temperature, the fluorescence intensity after 0, 30, 60, 120, and 240 min was measured. No decrease in fluorescence intensity (mean of *n* = 2 at each point) was observed. In contrast, the concentration of ozone in water measured using the ultraviolet absorption method decreased by half at 30 min after preparation of the ozone-containing water and completely disappeared after 120 min, as shown in Figure 8. Therefore, ozone can be measured with high reproducibly using probe** 1**.

## 4. Conclusion

Probe** 1** clearly reacted with ozone quantitatively and specifically and resulted in the formation of a stable fluorescent substance under the conditions described above. This assay is therefore useful for quantifying ozone in environmental science, biochemistry, and clinical chemistry by specifically detecting ozone, unlike conventional, nonspecific assays. This methodology may be useful in the quality control of ozone products and ozonizers.
